# The Degeneration and Regeneration of the Nerves

**Published:** 1864-12

**Authors:** 


### THE DEGENERATION AND REGENERATION OF THE NERVES.

M. Vulpian has lately discoursed on this very interesting subject in his lectures at the Museum of Natural History in Paris. The physiological property of motor and sensitive nervous fibres is the property of undergoing certain modifications under the influence of some agent. This property belongs to the nerve-fibre, independent of the nervous centers. "I am thoroughly convinced," says M. Vulpian, "that the origin of this property of the nerve is to be sought for in the nerve-fibre itself. Our classical works tell us that the nerves borrow their force or property from the nervous centres; but this is a complete error. If a motor nerve received its properties from the spinal marrow, it ought to lose them when cut; but it does nothing of the kind; for when the peripheric end of the cut nerve is excited, the muscles contract; and, more than this, when its nerve-force has been exhausted by long and continued excitation, it recovers its force under repose. It is strange that this simple demonstration has not convinced every one.

In considering the phenomena attending the disappearance of the nerve-force, we have to observe—1. The duration of the excitability of the nerve after section; 2. The mechanism of its disappearance.

In 1838, Muller investigated the first of these questions—the duration of the excitability of the nerve; and he concluded that it disappeared after some weeks. In 1840, Gunther and Schon made similar observations; and in 1841, M. Longet obtained similar results. It was also noted, that when, after section, the nerve had lost its properties, the muscle to which it was distributed still preserved its power of contractility—showing the independence of muscular irritability. Brown-Séquard and Martin Magron have seen muscular irritability last longer than two years in certain animals, although every trace of excitability had disappeared in the nerve which had been cut and passed into the muscles. Another proof of the fact that muscular contractility is independent of nervous excitability has been given by M. Bernard. He has demonstrated that, when the action of the motor nerves over the muscles is absolutely arrested, muscular irritability, contractility may still exist.

The question as to how the nerve loses its properties is of the highest interest, and its answer will give us a key to some of the most interesting phenomena which have exercised the sagacity of physiologists.

The force or property of the nerve disappears as a consequence of alteration of the nerve-substance; but this alteration is not appreciable by our means of investigation until the nerve-force has completely disappeared. The changes of degeneration which go on in the cut nerve are influenced by different circumstances. Thus the changes go on more rapidly in a young man than in an old animal. In the young, the complete change is effected in about two months; but in an old animal, not before six or seven months. The species of animal and season of the year also modify the result.

According to M. Waller, the change of structure of the nerve is due to interference with the nutrition of the nerve-fibres. The spinal marrow, in his view, is the centre of nutrition of the nerves—of the motor nerves, at least; so that, when the nerve is cut, its nutrition is disturbed, and change in structure results. M. Waller cut both the roots of a spinal nerve; and he found that change of structure did not occur in both of the so cut nerves. In the anterior root, he found the peripheric end alone degenerated; and in the posterior root, the central end. Hence he drew the conclusion, that the sensitive fibres have for their nutritive centre the ganglions of the posterior roots. This deduction is strengthened by the fact that, when the posterior root was ligatured beyond the ganglion, the outer end—the peripheric—was altered in structure.

On the same basis of facts, M. Schiff has made many interesting researches concerning recurrent sensibility. He cut the anterior root, and found, as Waller had done, that the peripheric nerve-fibres were changed, and that the central ones remained healthy. But he also found that some of the fibres in the peripheric cut end of the nerve remained sound; and that some in the central end were altered. And of these fibres, those which remained unchanged in the peripheric end, and those which were altered in the central end, were evidently fibres emanating from the posterior root. Hence the conclusion that recurrent sensibility is due to recurrent nervous fibres. How the grey substance can have a nutritive influence over the anterior root, and the ganglion over the posterior root, remains to be shown; but the fact appears certain.

In this way Waller has discovered an excellent means of studying the distribution of nerves by alteration of their fibres, and of recognizing in a mixed nerve the fibres which are sensory, and those which are motor. To this method of observation Waller has given the name of "new anatomical method."

We may, indeed, expect great results from this "method" of observation. Thus, for example, we know the union of the spinal accessory with the pneumogastric nerve. Well, if we divide the roots of the spinal accessory, wherever we find in the divisions of the pneumogastric altered nerve-fibres, we may safely say that they are fibres of the spinal accessory. If, again, we wish to know whether the nervus petrosus is a branch of the facial or of the fifth pair (through the pheno-palatine ganglion), we cut the facial nerve, and examine the petrous nerve, in the course of ten to fifteen days. We then find in it a mixture of healthy and changed nerve-fibres, and from this fact are justified in concluding that the petrosal nerve has a double origin. Again, does the chorda tympani go to the tongue? No; because, after cutting the facial, there is not found in the lingual a single fibre changed, beyond the fibres the submaxillary ganglion furnishes to the gland. These are examples of the value which this new method of observation renders to physiology and pathology.—*British Med. Journal.*

